# Mixed Linear Model Approaches of Association Mapping for Complex Traits Based on Omics Variants

**DOI:** 10.1038/srep10298

**Published:** 2015-07-30

**Authors:** Fu-Tao Zhang, Zhi-Hong Zhu, Xiao-Ran Tong, Zhi-Xiang Zhu, Ting Qi, Jun Zhu

**Affiliations:** 1 Institute of Bioinformatics, Zhejiang University, Hangzhou, China

## Abstract

Precise prediction for genetic architecture of complex traits is impeded by the limited understanding on genetic effects of complex traits, especially on gene-by-gene (*GxG*) and gene-by-environment (*GxE*) interaction. In the past decades, an explosion of high throughput technologies enables omics studies at multiple levels (such as genomics, transcriptomics, proteomics, and metabolomics). The analyses of large omics data, especially two-loci interaction analysis, are very time intensive. Integrating the diverse omics data and environmental effects in the analyses also remain challenges. We proposed mixed linear model approaches using GPU (Graphic Processing Unit) computation to simultaneously dissect various genetic effects. Analyses can be performed for estimating genetic main effects, *GxG* epistasis effects, and *GxE* environment interaction effects on large-scale omics data for complex traits, and for estimating heritability of specific genetic effects. Both mouse data analyses and Monte Carlo simulations demonstrated that genetic effects and environment interaction effects could be unbiasedly estimated with high statistical power by using the proposed approaches.

Both natural and experimental populations harbor an array of phenotypic variations because of the complicate genetic architecture underlying quantitative traits. It is well documented that the genetic basis responsible for phenotypic variability consists of individual causal genes and interacting networks, with their specific effects in multiple environmental conditions. Gene-by-gene (epistasis or *GxG*) and gene-by-environment (*GxE*) interactions, such as chicken comb type^1^, animal coat color, and the ABO blood group in humans, are confirmed to exist^2^. Complex traits are controlled by multiple loci, which harbor polymorphisms that give rise to phenotypic variation in a population. Complex traits cannot be studied by testing a single locus at a time, especially when the contribution of each locus is small^3^. To understand the genetic architecture of variation for complex traits, we need to perform system level analyses that encompass genome-wide SNPs, transcripts, proteins, and metabolites by considering the effects of *GxG* and *GxE* interactions.

In the past decades, an explosion of new high throughput technologies enables omics studies at multiple levels (such as genomics, transcriptomics, proteomics, and metabolomics). At each level it is possible to construct interaction networks associated with complex traits (including diseases)^4^. These large-scale omics data provide great opportunity for biological understanding, but integrating the diverse omics data and environmental effects in the analyses has remained a challenge. New computational methods need be developed to understand these complex heterogeneous omics data^5^,^6^,^7^,^8^,^9^. The analysis of large omics datasets, especially two-loci interaction analysis, involves intensive computation. Heterogeneous computational environments including graphic processing units (GPUs) system can provide effective solutions for large-scale data sets analysis^10^. CPU-GPU heterogeneous parallel computing is very common nowadays.

Linkage analyses and association analyses are two genetic mapping approaches used to assess the relation between the genotypic and phenotypic variations on a population scale. Taking advantage of conventional molecular markers, efficient statistical methods of QTL (Quantitative Trait Locus) mapping have become pervasive^11^ since the landmark approach (interval mapping) developed by Lander and Botstein^12^. Since then, several methods have been developed for searching epistasis^13^,^14^,^15^,^16^,^17^ and *GxE* interactions^18^,^19^,^20^,^21^. Mixed linear model-based composite interval mapping (MCIM)^22^,^23^,^24^ could detect both *GxG* and *GxE* interactions by experimental data involving multiple environments (or treatments). However, with the recent development of high-throughput genotyping technologies, genetic association analyses have become common tools for uncovering causal genetic variants and networks at the whole-genome level^25^. In 1947, Fisher first used linkage disequilibrium (LD) information to map casual loci for human blood types^26^. So far, many mapping studies of human diseases and complex traits by genetic association analyses have revealed plenty of novel loci and provided insight into the biology of diseases. Several methods have been published for exhaustive epistasis analysis^27^,^28^,^29^,^30^,^31^. However these methods cannot integrate other omics data except genome data. Because associating DNA (Deoxyribonucleic Acid) polymorphism with phenotypic variation omits all of the intermediate steps in the chain of causation from genetic perturbation of variation in quantitative traits, the intermediate molecular variables such as transcript abundance could allow us to interpret the causal networks^32^. The RNA expression microarray has been combined with other experimental approaches to find the key mechanism of complex traits^33^. One such technique considers the transcript abundance as a quantitative trait, known as expression quantitative trait locus (eQTL)^34^. Other approaches are to identify significantly expressed transcripts underlying complex traits by using a Pearson correlation coefficient^35^ and multiple linear regression^36^, in which the *GxG* and *GxE* at transcript levels are ignored. Despite intensive efforts to explain genetic variation of quantitative traits, which have identified a great number of genetic variants and transcripts for various complex traits, we still fall short of understanding the mechanism of the genetic architecture of complex traits.

In this study, mixed linear model approaches are proposed to identify genetic effects of individual loci, epistasis effects of pair-wise loci (Fig. 1a), as well as *GxE* interaction (Fig. 1b), which is applicable for genome-wide association studies (GWAS). Our approaches consist of four steps in statistical analyses: (1) one-dimension search for individual loci; (2) exhaustive two-dimension search for epistasis loci; (3) stepwise search for fitting a full genetic model, including candidate loci with main effects, epistasis, and *GxE* interaction; and (4) estimating gene effects of individual and epistasis loci detected in previous process by method of Monte Carlo Markov Chain via Gibbs Sampling^24^,^37^. All these processes have been implemented in a GPU-based mapping software, named QTXNetwork. With the massive parallel nature of multi-GPUs, association analyses can be performed for detecting loci on large-scale omics data for complex traits, and for estimating variance components of genetic effects. QTXNetwork consists of three functional modules: quantitative trait locus (QTL)^38^ for QTL analyses (Fig. 1c), quantitative trait SNP (QTS) for genome analyses and quantitative trait transcript/protein/metabolite (QTT/P/M) for transcriptome, proteome, or metabolome analyses (Fig. 1d). Association analyses can also be conducted for networks among four omics variants (Quantitative Trait X for SNPs, Transcripts, Proteins, and Metabolites) (Fig. 1e). By analyzing mouse datasets on anxiety and Monte Carlo simulations for linkage mapping of QTLs, association mapping of QTSs and QTTs, we demonstrated that unbiased estimation could be obtained for genetic effects of causal genes. The package QTXNetwork can be downloaded at the following website http://ibi.zju.edu.cn/software/QTXNetwork.

## Results

### Analysis of mouse data

We applied our proposed statistical methods for mapping QTLs, QTSs, and QTTs to searching for the genetic mechanism of anxiety in 71 BXD recombinant inbred (RI) strains of mice (*n* = 528 mice). Differences in the phenotypes are evident in the parental strains. For example, the maternal strain C57BL/6J exhibits lower anxiety- and fewer stress-related effects than the paternal strain DBA/2J, which exhibits greater fear-related responses^39^. Animals of 71 BXD RI strains, 60 to 120 days old, were used. These strains were derived by crossing C57BL/6J (B6) and DBA/2J (D2) strains in the 1970s (BXD1-32; 26 strains) and 1990s (BXD33-42; 9 strains)^40^. Genotypes of the BXD strains were generated at the University of Tennessee Health Science Center. A total of 3795 markers covering 19 autosomal chromosomes and one sex chromosome were genotyped, including 3,033 SNPs and 762 SSRs (Simple Sequence Repeats). Many adjacent markers had identical strain distribution patterns. Therefore, we selected 2,320 markers for the subsequent analysis (1,814 SNPs and 506 SSRs). On the other hand, there were 46,643 transcripts in total. Because many of them appeared to show no or little variation, we selected 4,193 transcripts with relatively large variance (coefficient of variation CV > 1.0%).

Anxiety-related behavior was examined in the closed quadrants of an elevated zero maze, a standard tool for testing anxiety^41^, under five conditions: 1) animals acutely restrained and receiving ethanol; 2) animals acutely restrained and receiving saline; 3) animals receiving only a saline injection; 4) animals receiving only an ethanol injection; and 5) animals not restrained or receiving any injection. Acutely restrained animals were placed in an immobilization tube for 15 minutes. Animals receiving injections were given either ethanol (1.8 g/kg) or saline and were returned to their home cages. The activities of the test session were recorded in the closed quadrants.

**Figure 2:**
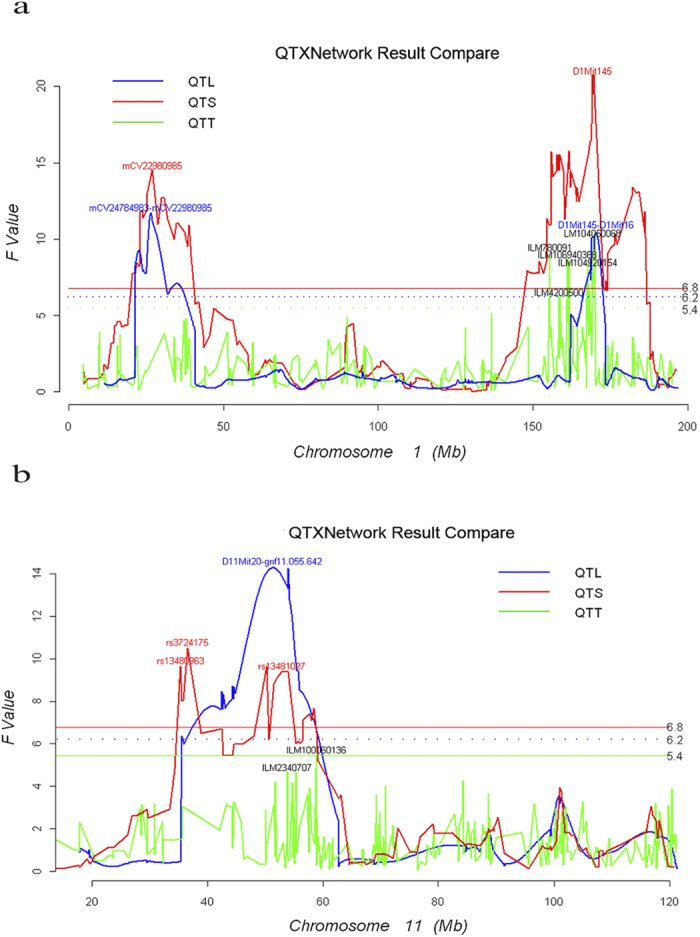
*F-*statistic plots from 1D genome scans by QTL linkage analysis, QTS and QTT association analysis on the 1^st^ chromosome (**a**) and the 11^th^ chromosome (**b**) (**a**) *F-*statistic plots from 1D genome scans by QTL, QTS, and QTT analyses on chromosomes 1. (**b**) *F-*statistic plots from 1D genome scans by QTL, QTS, and QTT analyses on chromosomes 11.

As shown in Fig. 2 and Table 1, there were three QTLs detected by linkage analysis on chromosomes 1 and 11, of which *Q*_1_ (within 25.2 Mb ~ 27.1 Mb) and *Q*_2_ (within 169.1 Mb ~ 169.8 Mb) were on chromosome 1, and *Q*_3_ (within 44.6 Mb ~ 53.9 Mb) was on chromosome 11. These three loci were confirmed by QTS association analysis with precision location (*Q*_1_ at 27.1 Mb, *Q*_2_ at 169.1 Mb, and *Q*_3_ at 52.8 Mb). Two extra QTS sites were also discovered on chromosome 11 (*Q*_4_ at 35.3 Mb and *Q*_5_ at 36.5 Mb). The QTS mapping matched well with exact position of identified SNP and higher power than QTL mapping. For the three loci detected by QTL and QTS mapping, only one was confirmed by QTT mapping (*Q*_2_ at 169.1 Mb), but another one was revealed nearby (*Q*_6_ at 155.5 Mb). It is apparent that QTT mapping can only discover transcript loci at the time when they are expressed.

As shown in Table 2, the epistasis loci *QQ*_1_ was identified with similar predicted effects by both QTL mapping (D1Mit291 × rs3659789) and QTS mapping (D1Mit291     × rs3717220). Compared with the QTL mapping, QTS mapping appeared to have higher statistical significance. Because no transcription *QQ*_1_ was detected on chromosome 1, there might have been no significant association of transcript epistasis *QQ*_1_ at the time when the tissue used for mRNA extraction was collected. There was another transcript epistasis *QQ*_2_ (ILM100060136 × ILM1740047) that was detectable only by QTT mapping.

### Monte Carlo simulations

A simulation study with 200 replications was conducted. The BXD mouse genetic map was used to generate three simulated populations for mapping QTLs, QTSs, and QTTs. Initially, we generated a simulated population for QTS mapping with 200 RIL genotypes consisting of 2,320 SNPs covering 2,037.6 cM. Five QTSs (denoted *Q*_1_, *Q*_2_, *Q*_3_, *Q*_4_, and *Q*_5_) were assumed to control the simulated trait. Four of the five QTSs were involved in the three pairs of two-way interactions, denoted *QQ*_1_ for *Q*_1_ × *Q*_3_, *QQ*_2_ for *Q*_1_ × *Q*_4_, and *QQ*_3_ for *Q*_3_ × *Q*_4_. The whole-genotype individuals were investigated in three environments. The individual SNPs and interactions were set to account for as much as 20% in total heritability (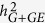). Detailed genetic information is listed in Table S2 and Table S3. For 200 simulations, we can detect significant individual QTLs/QTSs and pair-wise epistasis QTLs/QTSs. Power (%) was calculated as the percentage of true loci significantly detected. Mean of estimated genetic effects and standard error (*SE*) were also calculated for inferring un-biasedness of estimation of genetic effects.

A second simulation population was generated for mapping QTLs, including 506 microsatellite markers drawn from the entire 2,320 markers within each observation sample. Other parameters had the same settings as described above. A third simulation population was created for mapping QTTs, including 200 genotypes, with each composed of 2,320 transcript loci, using the same map as the mouse genetic map. Four transcript loci (denoted *Q*_1_, *Q*_2_, *Q*_3_, and *Q*_4_) were supposed to control the phenotype variation. Meanwhile, three pairs of two-loci combinations (denoted *QQ*_1_, *QQ*_2_, and *QQ*_3_) between the four transcript loci were assumed to be associated with the simulated trait. The 200-genotype individuals were tested in three environments. The total heritability was equal to 20%. Detailed information is listed in Table S4 and Table S5. Power of detecting loci and estimated genetic effects with their standard error (*SE*) were also calculated as for QTLs/QTSs mapping.

The Monte Carlo simulation demonstrated that mixed linear model approaches could robustly estimate positions and effects for QTLs, QTSs, and QTTs. The simulation results of mapping QTLs and QTSs are listed in Table S2 and Table S3. Our simulation results revealed that both QTL and QTS mapping approaches could obtain efficient and unbiased estimations of locations and genetic effects of loci with high power (>82.5% for individual loci and >87.0% for pair-wise epistasis loci). For example, *Q*_1_ (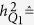 2.33%) and *Q*_4_ (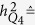 3.63%) had statistical power of 100% by both two methods. The loci with relatively small heritability may be more likely to be identified by QTS association analysis. Individual loci *Q*_5_ (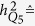 1.31%) had the smallest heritability among the simulated loci, which was detected with a statistical power of 90.5% by QTS association analysis, but only 82.5% by QTL linkage analysis. Similarly, for the locus *Q*_2_ with a heritability of 1.77%, QTS association analysis had higher statistical power (100%) than QTL linkage analysis (95%). Furthermore, the positions and genetic effects could be estimated more precisely by QTS association analysis. For a locus with a relatively large effect, both methods could yield an unbiased estimate. However, there were obvious differences between the two approaches for estimating genetic effects and positions of loci with relatively small heritability. For locus *Q*_5_, the smaller standard error (*SE*) of the estimated position indicated that QTS association analysis could define a more precise position than QTL linkage analysis. Because of the precise identification of position, the estimated effects of the locus may be closer to the parameters by the QTS association method. The estimated additive and additive-by-environment interaction effects of locus *Q*_5_ were also relatively accurate by QTS mapping. Likewise, the more precise estimation and smaller *SE* of the general additive effect of individual locus *Q*_4_ revealed that QTS mapping (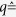 3.66, *SE* = 0.56) could obtain more accurate estimates than QTL mapping (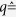 3.13, *SE* = 1.13).

Detailed simulation results for mapping QTTs are listed in Table S4 and Table S5. Association analysis of QTTs could also efficiently detect the casual transcript loci and provide unbiased estimations, such as positions, genetic main effects, and *GxE* interaction effects. Individual transcript loci could be detected with statistical power higher than 83.0%, and the power for detecting epistasis was 100% in all cases. The estimates of genetic effects and environment interaction effects were close to the parameter setting with very small *SE*s for individual transcript loci as well as two-transcript loci interactions. Because QTT association analysis could identify the transcript loci efficiently, we could obtain unbiased estimates of QTT main effects and QTT by environment interaction effects.

### GPU Accelerating Performance

We used three GPU servers to test the performance. The first one consisted of 2 NVIDIA GTX480 cards running on an Intel® core™ i7 × 980 with 3.33 GHz (Gigahertz) CPU using 12 GB (Gigabyte) DDR3 host memory. The second one consisted of 4 NVIDIA GTX680 cards running on an Intel® core™ E5645 with 2.40 GHz CPU using 48GB DDR3 host memory. The third one consisted of 4 NVIDIA Tesla K20c cards running on an Intel® core™ E5645 with 2.40 GHz CPU using 48GB DDR3 host memory. We compared the running time of three implementation versions, and measured the time of the whole procedure including the input, one-dimension search, two-dimension search, effect estimation, and the output as the comparing time. We implemented multi-GPU computing in two-dimension search. First we divided the whole SNP pairs into parts according to the number of GPUs and assigned each part to one GPU. Each GPU finished its tasks in loops. The speed-up results of GPU implements over single-thread CPU implementation are summarized in Table S6. We can see that the speed-up increases as the SNP number increasing. Given the same GPU architecture, the speedup is nearly in proportion to the number of GPUs. We can achieve more than 250 times speed-up by using four Tesla K20c cards. We used bit compression in QTS to save the memory space. We also tested the performance of GPU implementation with bit compression technology. Table S7 shows the speed-up of GPU implementation with compression over the single-thread CPU implementation. From Table S6 and Table S7 we can see that compression technology increased the performance instead of decreasing it. This was mainly because 1) we used bitwise operations instead of arithmetic operations to compress and decompress the data; 2) one GPU memory access can get more data by the compression. Therefore, one memory access can serve more GPU threads, and the number of memory access decreased. We have also used the newly developed software to analyze publicly available data (humans and plants) and detected major genetic variation due to dominance and epistasis for human BMI^42^, but epistasis and their environment interaction for cotton yield^43^.

## Discussion

Traditionally, linkage analyses can detect the causal individual QTLs and epistasis. Linkage mapping has discovered many QTLs affecting various quantitative traits. Because of the recent development of high-throughput genotyping technologies and identification of highly dense SNPs^44^, SNP markers have been commonly used in genome research^45^, bioinformatics and bio-computation studies^46^, genetic study of complex traits^47^, and population genetics of human beings^48^. As compared with linkage analyses, association analyses based on SNP markers have several advantages. Firstly, the QTS association mapping can be applied in different populations. QTL linkage mapping is realized by determining the probability of three genotypes (*QQ, Qq*, and *qq*), supposing the existence of linkage between the flanking markers and the unobserved loci. However, in artificially generated lines such as recombinant inbreeding lines (RILs) or doubled haploid lines (DHLs) derived from two parental lines, the abundant recombination may eliminate linkage over generations. Besides, it may be difficult to infer the probability of three genotypes in mapping QTLs for populations derived from multiple parental lines. The QTS association analyses rely on the retention of adjacent DNA variants over many generations. As a result, it is appropriate to detect loci for natural populations and complicated experimental designs by QTS association analyses.

For advanced populations, such as recombinant inbred lines (RILs) and near-isogenic lines (NILs), the linkage between the flanking markers and unobserved markers is reduced, as a few generations increase the recombination frequency^49^. This change may decrease the statistical power for detection of QTLs by the linkage analyses, because the reduced linkage may influence the prediction of three genotypes’ probability. On the contrary, because of the high density of SNP markers and observed genotypes, the association methods can detect QTSs efficiently, even QTSs with small heritability. From the results of simulations, it is revealed that the association analyses have higher statistical power than the linkage analyses, especially for loci with small heritability, such as *Q*_2_ and *Q*_5_ in Table S2. As shown in Fig. 2, higher peaks suggest that candidate loci may be detected more certainly by QTS association mapping. Furthermore, the candidate gene regions identified by QTL mapping may be large, encompassing hundreds or even thousands of genes. By contrast, the association analysis, drawing from historic recombination, may narrow the trait-associated regions to only one gene or gene fragment. In the Monte Carlo simulations, the individual QTL *Q*_5_ in Table S2 had the smallest heritability. The QTS association analyses obtained smaller *SE*s of estimated position than the QTL linkage analyses. In addition, when analyzing the data of the mouse on chromosome 11, the QTS association mapping detected two significant SNPs in the region of the QTL mapped by the linkage study. It is revealed that QTS association analysis has advantages over linkage analysis for efficiency and accuracy in mapping loci.

Discovered loci such as QTSs can subsequently be used to predict phenotypic values and QTS effects in an independent population, and it typically provides some improvement in classifying phenotypic values over random decision-making. In public health, it is useful to determine whether individuals are in an at-risk group. Owing to the accuracy of locus position and effect estimation, and the ease of discovery of loci with low heritability, the effective and efficient QTS association can improve the genetic predictor.

On the other hand, transcript association can detect causal transcript loci efficiently. In contrast to QTS association analysis and QTL linkage analysis, the genotypic variants of QTT association are continuous gene expression data. The high statistical power and unbiased estimation indicates that QTT association is also a useful approach to map individual transcripts and pair-wise interaction, which are significantly associated with the quantitative traits. In addition, the approach could also be extended to mapping quantitative trait protein (QTP) and quantitative trait metabolite (QTM)^50^. Combining the results of transcript association with the QTSs mapped by association analyses, we could further understand the function of the candidate genes. Although we detected several loci by the linkage analyses and association analyses, they may affect the quantitative traits by a specific unknown mechanism. We can settle the problem by QTT association analyses. For example, in the case of anxiety of the mouse, we found three individual loci by both QTL linkage mapping and QTS association mapping. The transcript association mapping shows that only one of them was associated at the gene expression level with anxiety. Thus, it is a useful approach to combine the intermediate molecular phenotypes with QTS mapping to understand the biologically causal networks. Moreover, as other intermediate molecular variations, such as proteins and metabolites, we can further explore the “black box” of complex traits.

## Methods

### Mixed linear model

For mapping quantitative trait SNP (QTS) or quantitative trait transcript/protein/metabolite (QTT/QTP/QTM), mixed linear model approaches can be used to detect loci significantly associated with phenotypic variation^51^,^52^,^53^,^54^,^55^. When quantitative variation of transcripts, proteins, and metabolites are used as independent variables for association analyses among these three omics genotypic variants, other types of QTXs can be identified. The names of total 16 types of QTXs detectable by association mapping are listed in Table S1.

Mixed-model approach for QTL mapping^24^,^37^ can deliver unbiased estimation of genetic effects (additive, dominance, epistasis and their environment interaction) for detected loci based on a genetic model with genetic main effects as fixed effects and environment interaction effects as random effects. For analyzing large amount of candidate omics variants by associating mapping, we proposed to use genetic model setting all genetic effects as random variables. For mapping SNPs in homozygote population and transcripts/proteins/metabolites in homozygote/heterozygote population, the dependent variables (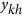) of the *k*-*th* subject in the *h-th* environment can be expressed by the following mixed linear model:
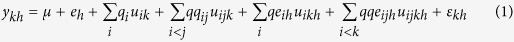
where 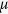 is the population mean; 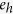
is the fixed effect of the *h*-*th* environment; 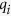
is the *i*-*th* locus effect with coefficient 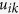
(1 for *QQ*, -1 for *qq,* and 0 for *Qq* in QTS mapping, and using expression values in QTT/P/M mapping); 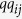 is the epistasis effect of locus *i* × locus *j* with coefficients 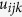
(1 for *QQ × QQ* and *qq × qq,* -1 for *QQ × qq* and *qq × QQ* in QTS mapping, and using expression values 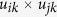 in QTT/P/M mapping); 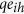 is the environment interaction effect of the *i*-*th* locus in the *h*-*th* environment with coefficient 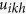; 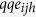 is the epistasis × environment interaction effect of locus *i* × locus *j* in the *h*-*th* environment with coefficient 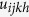; and 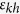 is the residual effect of the *k*-*th* individual in the *h*-*th* environment.

The mixed linear model can be presented in matrix notation:
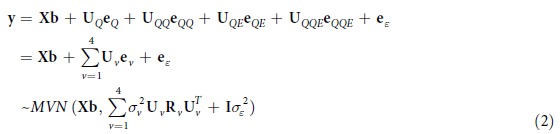
where 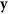 is an *n* × 1 column vector of phenotypic values and *n* is the number of sample observations; 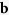 is a column vector of μ and environment effects; 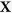 is the known incidence matrix relating to the fixed effects; 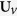 is the known coefficient matrix relating to the *v*-*th* random vector 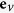; 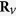 is the kinship coefficient matrix relating to the *v*-*th* random vector 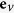; and 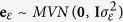 is an *n* × 1 column vector of residual effects.

To identify the susceptible individual and epistasis loci, we can conduct two-step approaches:

1. Individual locus detection. To test significance of the *i-th* individual locus, we used the following mixed linear model
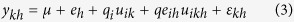
where the parameters are defined as in Equation (1). We performed the *F*-test step by step based on the Henderson method III^56^. The locus with maximum *F*-value^24^ exceeding a predefined critical value (experiment-wise error rate 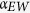 < 0.05) is considered as a candidate individual SNP or transcript.
2. Epistasis loci detection. In order to search all possible epistasis interacting loci when s individual locus has been selected by the first step, we conduct an exhausted two dimension (2D) genome scan by the following statistical model.
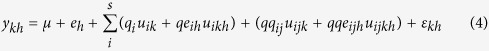

where the parameters have the same definitions as in Equation (1). The *F*-test is performed to test all possible pairs. The pairs of loci with maximum *F*-value larger than the predefined threshold value (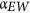 < 0.05) are considered as candidate epistasis interacting loci.

After selecting the candidate individual and pair-wise loci, a full statistical model as in Equation (1) is used to estimate variance components and genetic effects by mixed linear model approaches. Variance components in the following equations can be estimated by MINQUE(1) method (Minimum Norm Quadratic Unbiased Estimation setting prior values as 1)
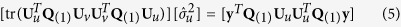
where
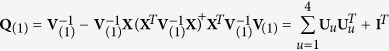


Genetic effects can be predicted by an Adjusted Unbiased Prediction (AUP) method^57^
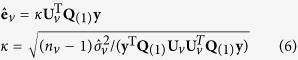


In the detection of individual and epistasis loci association with the phenotypic variation, multiple hypothesis tests are conducted among the candidate genotypes. To control experiment-wise type I error, a permutation testing is applied. Because the statistical model consists of parameters to be tested for putative individual loci in a two-locus detection process, we randomly shuffle the order of parameters to be tested. 2000 permutations were used to calculate the critical *P*-value for controlling the experiment-wise type I error. Stepwise selection was performed on all the significant peaks selected from the *F*-statistic profile, which meets the significance level (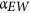 < 0.05) of experiment-wise type I error^24^,^37^. The effects of individual and epistasis interacting loci detected in the previous process are estimated by the following mixed model equations via Markov chain Monte Carlo (MCMC)^24^,^37^:
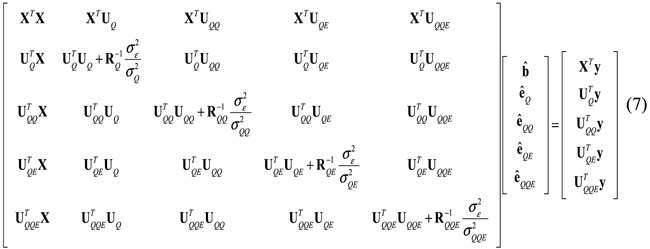


In the current study, a chain length of 200,000 and a thinning interval of 10 cycles were employed for parameter to be estimated, after the chain reached the equilibrium distribution.

### GPU Computing Implementation

We implemented mixed linear model approaches with architecture of CPU-GPU heterogeneous parallel computation. The designing of QTT/M/P mapping is similar to QTS mapping. For illustrating how computation is performed, we took QTS mapping as an example and drew Fig. S1 showing the computational flow chart. We exploited GPU computing on one-dimension search for individual loci and two-dimension search for epistasis loci, which are the most time-consuming steps among the whole statistical analyses. Other less time-consuming statistical analysis steps and the input/output procedure ran on CPU. Moreover, a self-adaptive load balancing method and a matrix compression method for coefficient matrix of mixed linear model were exploited. In order to hide the GPU latency, the number of running warps (32 threads a warp) on SM (Stream Multiprocessor) should be set as many as possible. In general the size of grid should be at least three times of the number of SM. Moreover there should be more than four warps in a Block. In one-dimension search and two-dimension search, we exploited one to one model. One candidate locus test or one interaction test is finished by one GPU thread.

In one-dimension search, the significance of one locus was analyzed by one GPU thread. In this step, some optimization technologies (Divide and Conquer, Coalesced Memory Access and Matrix Compression) were exploited. The framework is shown as Fig. S2.

In two-dimension search, one pair of loci was tested by one GPU thread. Because of the high throughput technology, the pair number can be very huge. We implemented the interaction scan on multi-GPU platforms. We have drawn Fig. S3 showing the framework of single GPU implementation and Fig. S4 showing the framework of multiple GPUs implementation. In two-dimension search scan, some data structures such as phenotype vector, permutation matrix and coefficient matrix should be copied from host memory to GPU global memory. Each interaction test has a different coefficient matrix. All these necessary coefficient matrices should be copied to GPU global memory. We used bit compression technology to compress these matrices. A lot of memory space and transfer time were saved. Besides this technology OpenMP, Divide and Conquer were exploited.

## Additional Information

**How to cite this article**: Zhang, F.-T. *et al*. Mixed Linear Model Approaches of Association Mapping for Complex Traits Based on Omics Variants. *Sci. Rep*. **5**, 10298; doi: 10.1038/srep10298 (2015).

## Supplementary Material

Supplementary Information

## Figures and Tables

**Figure 1:**
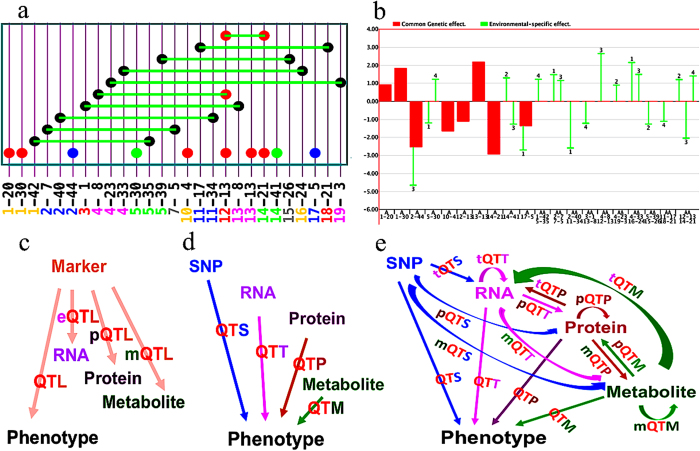
A combined platform for linkage and association analyses (**a**) *GxG* plot generated by QTX mapping. Circle=additive effect locus; Line between two circles=epistasis effect of two loci; Red color=main effect; Green color=environment-specific effect; Blue color=both main and environment-specific effects; Black color=involving epistasis but with no individual locus effect; (**b**) *GxE* plot generated by QTX mapping. The left axis is the values of genetic effects, and the bottom axis is the SNP ID for loci; Red column=main effect, green line=environment-specific effect; A=additive effect; AA=additive-by-additive epistasis effect; (**c**) Linkage mapping for quantitative trait loci of independent variants of phenotype (QTL), transcript (eQTL), protein (pQTL), and metabolite (mQTL). (**d**) Association mapping for phenotypic variation due to independent variants of quantitative trait SNP (QTS), quantitative trait transcript (QTT), quantitative trait protein (QTP), and quantitative trait metabolite (QTM). (**e**) Association mapping for different independent variables to dependent variables among phenotypic and 4 omics variants.

**Table 1:** 

Method	Chromosome (Position, Mb)	SNP Name	*q*	*qe*_1_	*qe*_2_	*qe*_3_	*qe*_4_	*qe*_5_
QTL	Q_1_: Chr1 (25.2-27.1)	mCV22980985	30.8‡	–18.7*	27.6‡	–19.8*	25.6†	
	Q_2_: Chr1 (169.1-169.8)	D1Mit145	–33.4‡					
	Q_3_: Chr11 (44.6-53.9)	rs13481018	30.6‡	–28.3†	35.3‡	–24.0**	29.5†	
QTS	Q_1_: Chr1 (27.1)	mCV22980985	4.2^+^					
	Q_2_: Chr1 (169.1)	D1Mit145	–19.3‡					
	Q_4_: Chr11 (35.3)	rs13480963			–41.6‡		36.5‡	
	Q_5_: Chr11 (36.5)	rs3724175	6.5*		30.1‡		–21.2**	19.0*
	Q_3_: Chr11 (52.8)	rs13481027			45.9‡		23.2†	
QTT	Q_6_: Chr1: (155.5)	ILM780091	61.3‡				–23.1‡	32.8‡
	Q_2_: Chr1: (169.1)	ILM104050068	45.5‡		29.4*	–18.0**		

Estimated positions and effects of individual loci detected by QTL linkage analysis, QTS and QTT association analyses. Note: q = additive effect of QTL and QTS, individual transcript loci effect of QTT; qe = locus by environment interaction effect; Signal after the effects, *
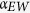
< 0.05, **
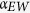
< 0.01, †
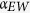
< 0.05, ‡
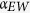
< 0.001.

**Table 2:** 

Method	Position (Mb)	Name	Position (Mb)	Name	*qq*	*qqe*_1_	*qqe*_2_	*qqe*_3_	*qqe*_4_	*qqe*_5_
QTL	QQ_1_: Chr1 (186.4-188.0)	D1Mit291	Ch8 (40.9-60.3)	rs3659789		23.0**	–40.2‡	25.9†	–41.7‡	33.6‡
QTS	QQ_1_: Chr1 (186.4)	D1Mit291	Ch8 (59.1)	rs3717220		20.1**	–20.0**	20.0**	–14.2^+^	32.5‡
QTT	QQ_2_: Chr11 (58.8)	ILM100060136	Ch14 (33.2)	ILM1740047	4.7‡	–1.0‡	3.0‡	1.1‡	5.0‡	–1.9‡

Estimated positions and effects of epistasis detected by QTL linkage mapping, QTS and QTT association analyses. Note: Signal after the effects, *, **, † and ‡ as defined in Table 1; *qq =* additive by additive effect; *qqe* = epistasis loci by environment interaction effect.
