# Supplementary material for: Mixed Linear Model Approaches of Association Mapping for Complex Traits Based on Omics Variants
Source: Sci Rep. 2015 Jul 30;5:10298. doi: 10.1038/srep10298 (PMC5155518; doi:10.1038/srep10298)

Supplementary Information

**Mixed Linear Model Approaches of Association Mapping**

**for Complex Traits Based on Omics Variants**

Fu-Tao Zhang*, Zhi-Hong Zhu*, Xiao-Ran Tong, Zhi-Xiang Zhu, Ting Qi and Jun Zhu

Institute of Bioinformatics, Zhejiang University, Hangzhou, China

*These authors contributed equally to this work

Correspondence and requests for materials should be addressed to J.Z. (jzhu@zju.edu.cn)

**Table S1. Definition of QTX for associating different dependent variables of trait to independent variables of 4 omics variants**

| **Dependent variable** | **Independent variable** | | | |
| --- | --- | --- | --- | --- |
|  | **Metabolite** | **Protein** | **RNA** | **SNP** |
| **(1) Phenotype** | **QTM** | **QTP** | **QTT** | **QTS** |
| **(2) Transcript** | **tQTM** | **tQTP** | **tQTT** | **tQTS** |
| **(3) Protein** | **pQTM** | **pQTP** | **pQTT** | **pQTS** |
| **(4) Metabolite** | **mQTM** | **mQTP** | **mQTT** | **mQTS** |

Note: (1) Associating phenotype trait to molecular variants of quantitative trait metabolite (**QTM**), quantitative trait protein (**QTP**), quantitative trait transcript (**QTT**), and quantitative trait SNP (**QTS**); (2) Associating transcript expression trait to molecular variants of quantitative trait metabolite (**tQTM**), quantitative trait protein (**tQTP**), quantitative trait transcript (**tQTT**), and quantitative trait SNP (**tQTS**); (3) Associating protein expression trait to molecular variants of quantitative trait metabolite (**pQTM**), quantitative trait protein (**pQTP**), quantitative trait transcript (**pQTT**), and quantitative trait SNP (**pQTS**). (4) Associating metabolite expression trait to molecular variants of quantitative trait metabolite (**mQTM**), quantitative trait protein (**mQTP**), quantitative trait transcript (**mQTT**), and quantitative trait SNP (**mQTS**).

**Table S2. Simulation results of estimating positions and effects for QTL and QTS mapping of individual loci.**

| Parameter | Method | *Q*_1_ | *Q*_2_ | *Q*_3_ | *Q*_4_ | *Q*_5_ |
| --- | --- | --- | --- | --- | --- | --- |
| (%) |  | 2.33 | 1.77 | 2.85 | 3.63 | 1.31 |
| Chromosome |  | 1 | 7 | 10 | 15 | 18 |
| Position (cM) |  | 56.34 | 39.61 | 44.65 | 26.81 | 62.43 |
| Estimates (*SE*) | QTL | 56.31 (1.83) | 39.87 (2.16) | 44.01 (2.09) | 26.75 (1.79) | 62.07 (3.83) |
|  | QTS | 56.14 (1.82) | 39.36 (2.07) | 44.56 (1.48) | 26.48 (1.56) | 62.76 (0.86) |
| *a* |  | –2.98 | –2.60 | 1.96 | 3.72 | 0 |
| Estimates (*SE*) | QTL | –2.52 (0.90) | –2.08 (0.91) | 1.31 (0.73) | 3.13 (1.13) | 0.02 (0.33) |
|  | QTS | –2.80 (0.81) | –2.58 (0.53) | 2.00 (0.49) | 3.66 (0.56) | –0.01 (0.12) |
| *ae*_1_ |  | 0 | 0 | –2.21 | 0 | 2.58 |
| Estimates (*SE*) | QTL | –0.00 (0.14) | –0.01 (0.20) | –1.25 (1.24) | –0.00 (0.13) | 2.33 (0.76) |
|  | QTS | –0.01 (0.10) | –0.04 (0.29) | –1.96 (0.80) | 0.03 (0.24) | 2.51 (0.64) |
| *ae*_2_ |  | 0 | 0 | –0.72 | 0 | –1.31 |
| Estimates (*SE*) | QTL | 0.02 (0.17) | 0.00 (0.00) | –0.13 (0.74) | 0.01 (0.14) | –0.70 (1.11) |
|  | QTS | –0.02 (0.19) | –0.03 (0.19) | 0.00 (0.73) | 0.06 (0.30) | –0.81 (0.94) |
| *ae*_3_ |  | 0 | 0 | 2.93 | 0 | –1.26 |
| Estimates (*SE*) | QTL | –0.01 (0.15) | –0.01 (0.08) | 3.42 (0.86) | 0.01 (0.15) | –0.69 (1.08) |
|  | QTS | –0.02 (0.18) | –0.01 (0.20) | 2.68 (0.65) | 0.03 (0.29) | –0.79 (0.95) |
| Power (%) | QTL | 100 | 95.0 | 94.0 | 100 | 82.5 |
|  | QTS | 100 | 100 | 93.0 | 100 | 90.5 |

Note: QTL = QTL linkage mapping analysis; QTS = quantitative trait SNP association analysis; False discovery rate = 0.0517 for QTL and 0.1049 for QTS; % = heritability of single locus; *a* = additive effect; *ae* = additive by environment interaction effect in different environment; *SE* = standard error.

**Table S3.** Simulation results of estimating positions and epistasis effects for QTL and QTS mapping of two loci.

| Parameter | Method | *QQ*_1_ (*Q*_1_×*Q*_3_) | *QQ*_2_ (*Q*_1_×*Q*_4_) | *QQ*_3_ (*Q*_3_×*Q*_4_) |
| --- | --- | --- | --- | --- |
| (%) |  | 2.02 | 3.42 | 2.67 |
| *aa* |  | 2.78 | 2.66 | 3.19 |
| Estimates (*SE*) | QTL | 2.56 (0.58) | 2.69 (0.64) | 2.92 (0.59) |
|  | QTS | 2.33 (0.78) | 1.87 (0.91) | 2.62 (0.91) |
| *aae*_1_ |  | 0 | –2.19 | 0 |
| Estimates (*SE*) | QTL | 0.00 (0.00) | –1.81 (1.09) | 0.01 (0.12) |
|  | QTS | 0.02 (0.19) | –0.97 (1.48) | 0.00 (0.00) |
| *aae*_2_ |  | 0 | 2.64 | 0 |
| Estimates (*SE*) | QTL | –0.02 (0.19) | 2.35 (0.97) | –0.01 (0.13) |
|  | QTS | 0.01 (0.18) | 2.86 (0.90) | 0.02 (0.16) |
| *aae*_3_ |  | 0 | –0.45 | 0 |
| Estimates (*SE*) | QTL | 0.00 (0.00) | –0.05 (0.48) | –0.00 (0.12) |
|  | QTS | 0.01 (0.08) | 0.03 (0.52) | 0.00 (0.00) |
| Power (%) | QTL | 93.5 | 88.5 | 94.5 |
|  | QTS | 87.5 | 87.0 | 92.5 |

Note: QTL = QTL linkage mapping; QTS = quantitative trait SNP association analysis; False discovery rate = 0.0595 for QTL and 0.166 for QTS; % = heritability of two-loci epistasis; *aa* = additive by additive effect; *aae* = *aa* by environment interaction effect in different environment; *SE* = standard error.

**Table S4.** Simulation results of estimating positions and effects for QTT mapping of individual loci.

| Parameter | *Q*_1_ | *Q*_2_ | *Q*_3_ | *Q*_4_ |
| --- | --- | --- | --- | --- |
| (%) | 2.67 | 3.82 | 2.15 | 3.10 |
| Chromosome | 1 | 7 | 15 | 18 |
| Position (cM) | 56.34 | 39.61 | 26.81 | 62.43 |
| Estimates (*SE*) | 61.15 (5.17) | 40.32 (2.15) | 27.03 (1.45) | 62.83 (0.89) |
| *q* | –3.32 | –2.87 | 2.98 | 2.60 |
| Estimates (*SE*) | –3.10 (0.73) | –1.56 (1.55) | 2.74 (0.87) | 1.84 (0.92) |
| *qe*_1_ | 0 | 2.82 | 0 | 0.51 |
| Estimates (*SE*) | –0.07 (0.23) | 1.43 (1.62) | 0.04 (0.21) | 1.27 (0.98) |
| *qe_2_* | 0 | –0.18 | 0 | 2.15 |
| Estimates (*SE*) | 0.00 (0.23) | –0.87 (0.97) | –0.01 (0.23) | 2.67 (0.93) |
| *qe_3_* | 0 | –2.64 | 0 | –2.66 |
| Estimates (*SE*) | –0.03 (0.24) | –2.84 (1.48) | 0.04 (0.28) | –1.80 (1.02) |
| Power (%) | 94.5 | 100 | 85.0 | 83.0 |

Note: False positive rate = 0.0213; (%) = heritability of single locus; *q* = individual transcript effect; *qe* = transcript by environment interaction effect in different environment; *SE* = standard error.

**Table S5**. Simulation results of estimating positions and epistasis effects for QTT mapping of two loci.

| Epistasis | *QQ*_1_ (*Q*_1_×*Q*_3_) | *QQ*_2_ (*Q*_1_×*Q*_4_) | *QQ*_3_ (*Q*_3_×*Q*_4_) |
| --- | --- | --- | --- |
| (%) | 2.76 | 3.38 | 2.13 |
| *qq* | 3.37 | 2.63 | 2.96 |
| Estimates (*SE*) | 2.29 (1.73) | 1.09 (1.69) | 1.83 (1.71) |
| *qqe*_1_ | 0 | 0.69 | 0 |
| Estimates (*SE*) | 0.16 (0.41) | 1.59 (1.27) | 0.11 (0.30) |
| *qqe*_2_ | 0 | 2.23 | 0 |
| Estimates (*SE*) | 0.16 (0.42) | 2.82 (1.46) | 0.04 (0.28) |
| *qqe*_3_ | 0 | –2.92 | 0 |
| Estimates (*SE*) | 0.12 (0.39) | –1.29 (1.80) | 0.08 (0.28) |
| Power (%) | 100 | 100 | 100 |

Note: False positive rate = 0.1392; (%) = heritability of two-loci epistasis; *qq* = epistasis transcript effect; *qqe* = epistasis by environment interaction effect in different environment; *SE* = standard error.

**Table S6. Speed-up of QTS on simulated data set (1200 observations)**

| GPU | 600 SNPs | 1200 SNPs | 1800 SNPs | 2400 SNPs |
| --- | --- | --- | --- | --- |
| GTX480 | 31.6 | 37.3 | 45.1 | 57.9 |
| 2×GTX480 | 47.9 | 66.9 | 85.4 | 110.6 |
| GTX680 | 33.8 | 49.3 | 48.6 | 69.4 |
| 4×GTX680 | 76.4 | 145.3 | 170.2 | 230.9 |
| Tesla K20c | 34.2 | 52.7 | 56.5 | 71.2 |
| 4×Tesla K20c | 77.1 | 167.3 | 171.8 | 252.4 |

**Table S7. Speed-up of QTS GPU implementation with bit compression on simulated data set (1200 observations)**

| GPU | 600 SNPs | 1200 SNPs | 1800 SNPs | 2400 SNPs |
| --- | --- | --- | --- | --- |
| GTX480 | 36.9 | 39.2 | 47.6 | 61.1 |
| 2×GTX 480 | 49.0 | 67.7 | 87.8 | 111.4 |


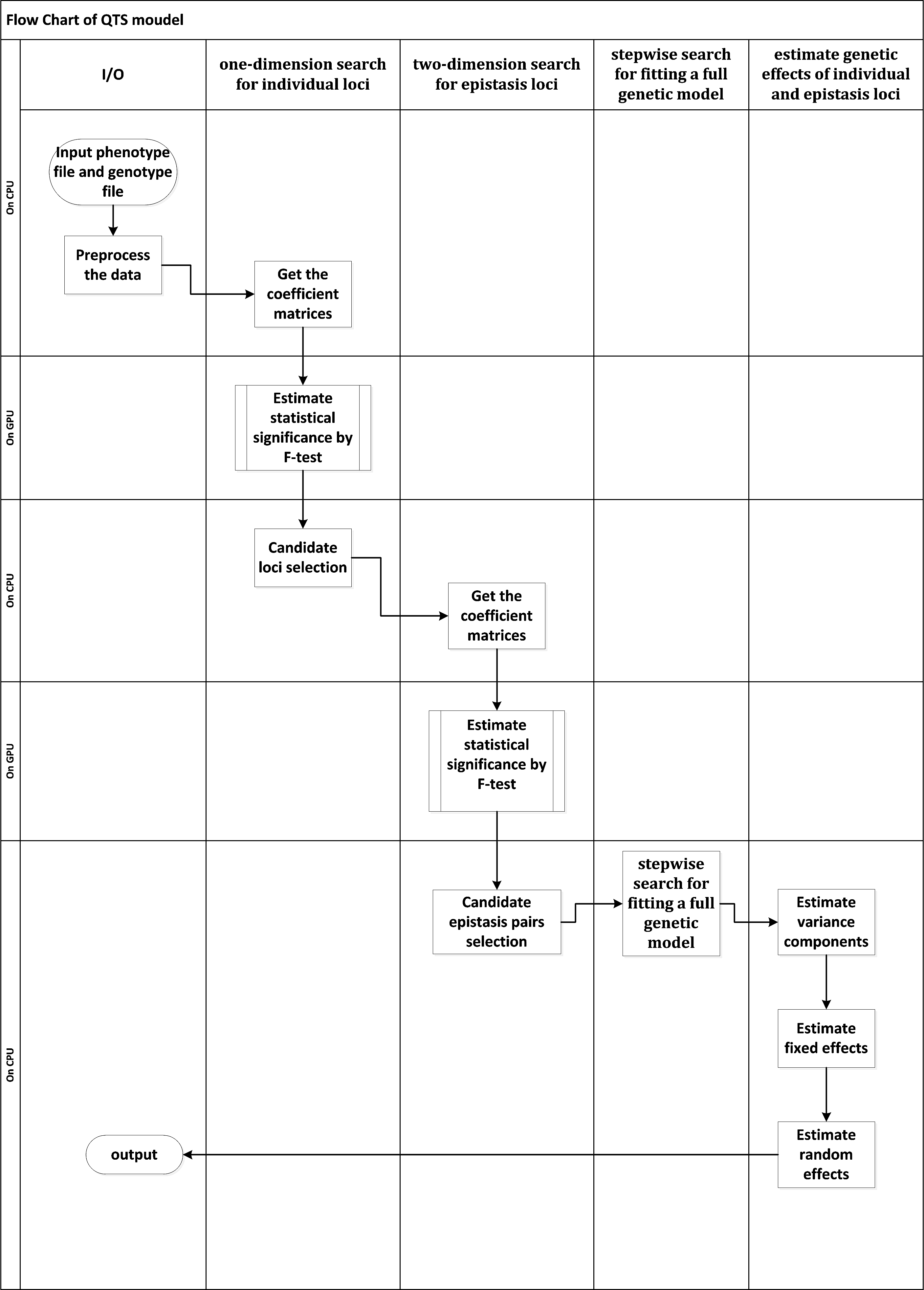


**Figure S1.** Computational Flow Chart of QTS

**Figure S2.** Framework of candidate loci scan


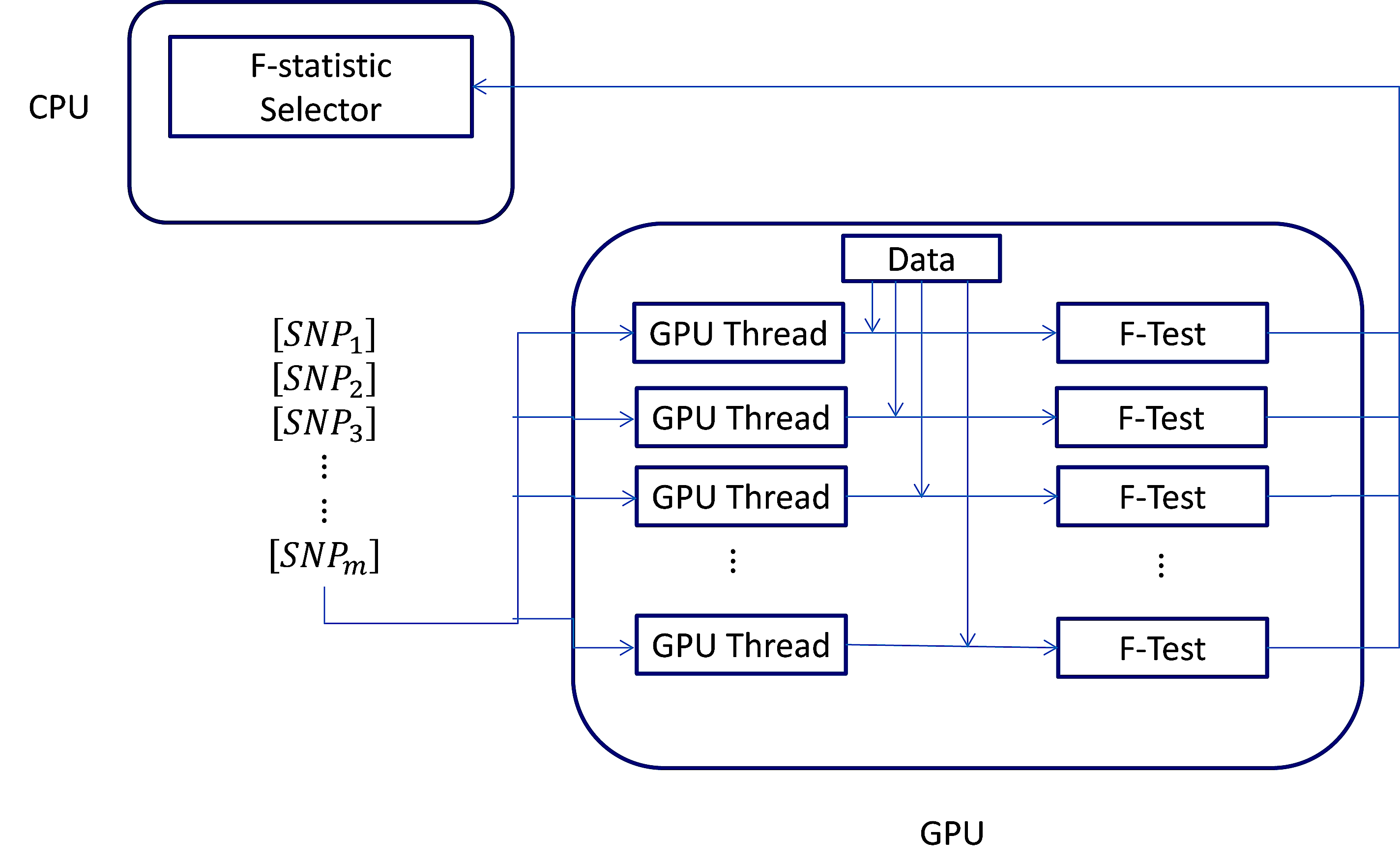


**Figure S3.** Framework of interaction scan on single GPU


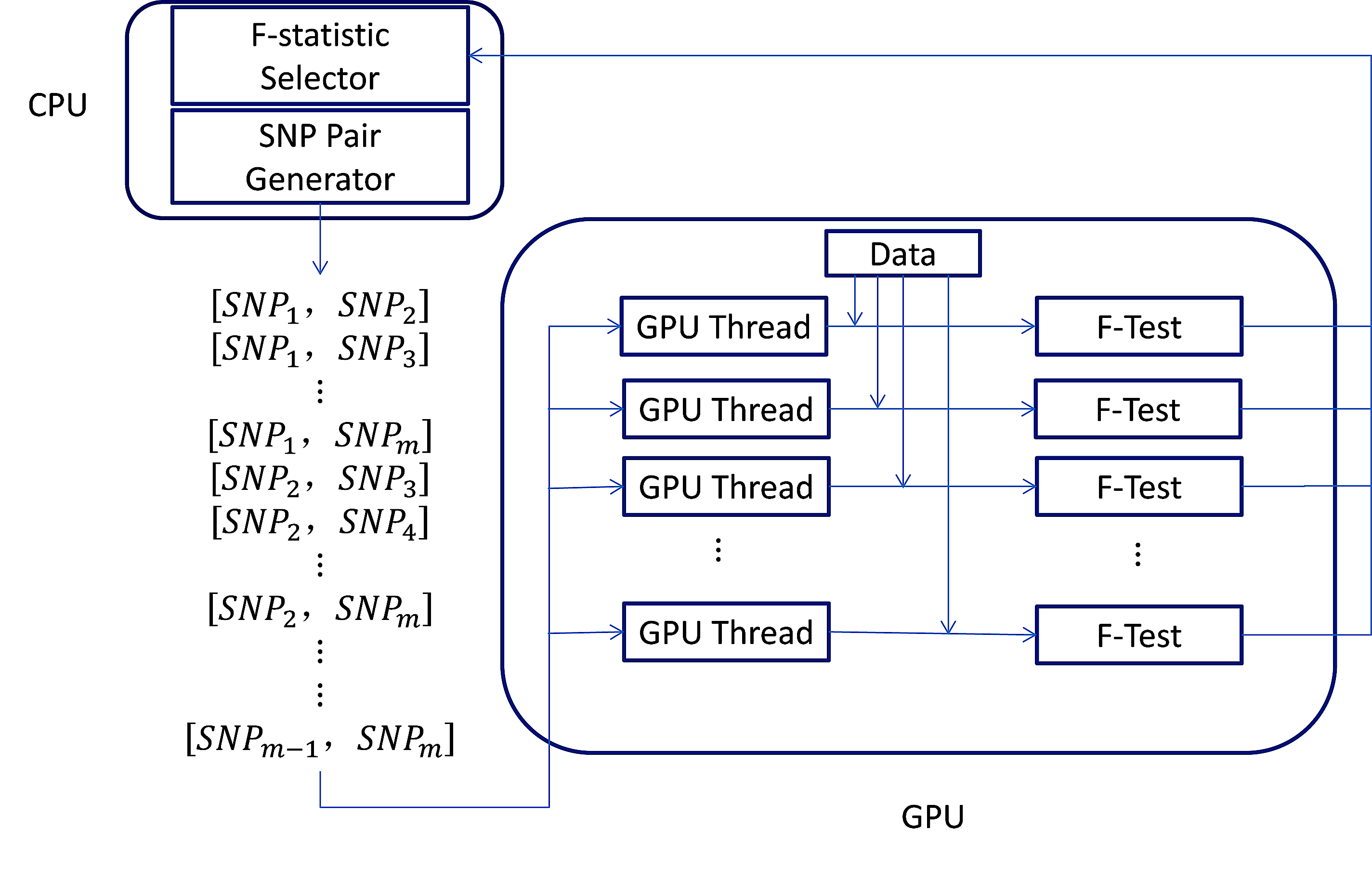


**Figure S4.** Framework of interaction scan on multiple GPUs


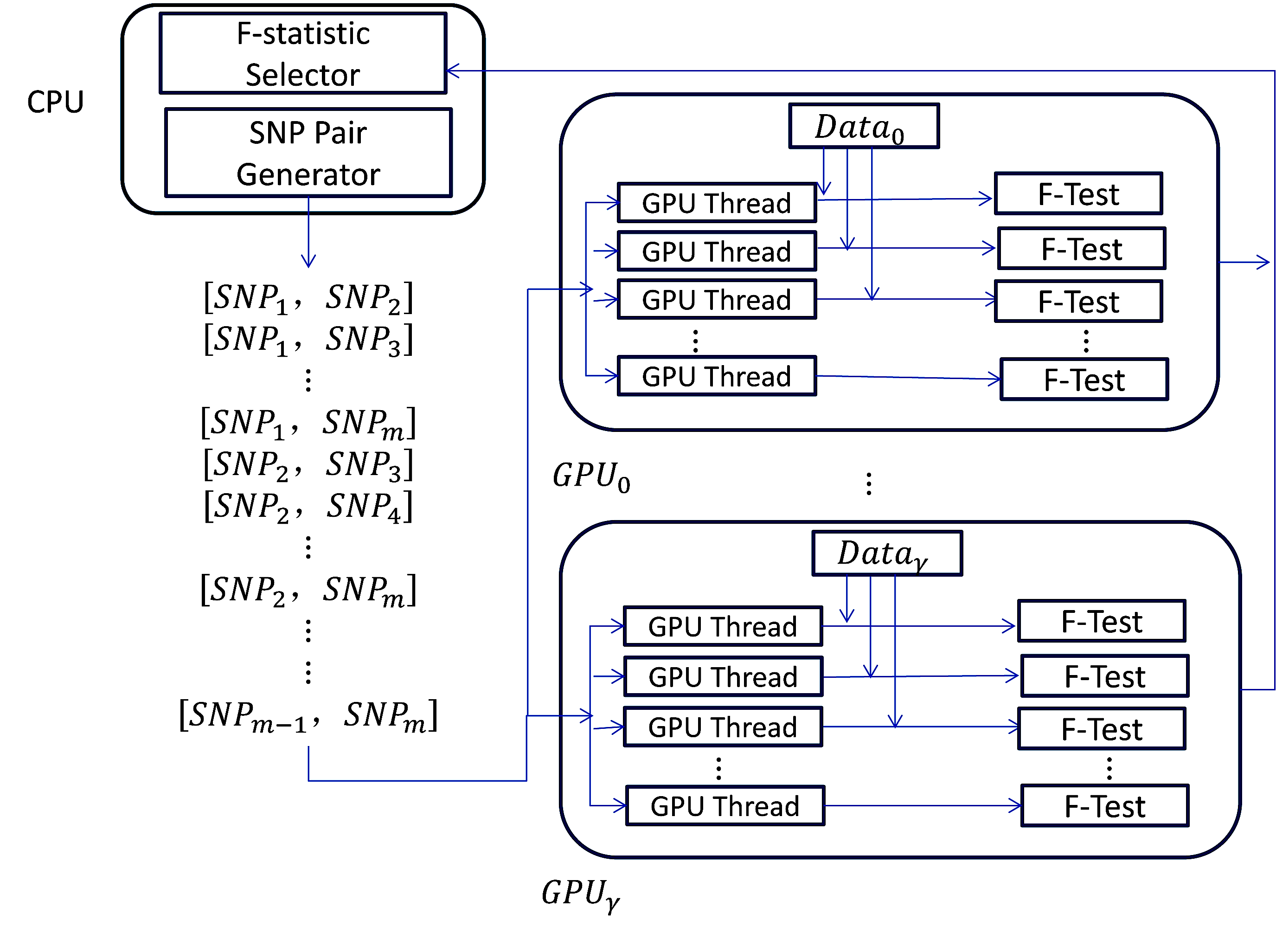

Supplement: Supplementary Information [file srep10298-s1.doc]
